# Associations of physical fitness with brain structure, pathology and cognition in cognitively normal older adults

**DOI:** 10.1038/s41514-026-00512-4

**Published:** 2026-09-16

**Authors:** Svenja Schwarck, Niklas Behrenbruch, Beate Schumann-Werner, Eóin N. Molloy, Berta Garcia-Garcia, Anne Hochkeppler, Larissa Fischer, Anna-Therese Büchel, Enise I. Incesoy, Jose Bernal, Roberto Duarte Coello, Maria d. C. Valdés-Hernández, Joanna M. Wardlaw, Niklas Vockert, Patrick Müller, Gusalija Behnisch, Barbara Morgado, Hermann Esselmann, Constanze I. Seidenbecher, Björn H. Schott, Henryk Barthel, Osama Sabri, Jens Wiltfang, Michael C. Kreissl, Emrah Düzel, Anne Maass

**Affiliations:** 1 German Center for Neurodegenerative Diseases (DZNE), Magdeburg, Germany; 2 Institute of Cognitive Neurology and Dementia Research (IKND), Otto von Guericke University Magdeburg, Magdeburg, Germany; 3 Faculty of Natural Science, Institute for Biology, Otto-von-Guericke-University Magdeburg, Magdeburg, Germany; 4 Faculty of Medicine, Division of Nuclear Medicine, Department of Radiology & Nuclear Medicine, Otto von Guericke University Magdeburg, Magdeburg, Germany; 5 Department of Neurobiology and Behavior, University of California, Irvine, CA USA; 6 Department of Neuroimaging Sciences, Row Fogo Centre for Research into Ageing and The Brain, Institute for Neuroscience and Cardiovascular Research, The University of Edinburgh, Edinburgh, UK; 7 Department Artificial Intelligence in Biomedical Engineering (AIBE), Friedrich-Alexander-Universität (FAU) Erlangen-Nürnberg, Erlangen, Germany; 8 UK Dementia Research Institute Centre at the University of Edinburgh, Edinburgh, UK; 9 Division of Cardiology and Angiology, University Hospital Magdeburg, Magdeburg, Germany; 10 Leibniz Institute for Neurobiology (LIN), Magdeburg, Germany; 11 Department for Psychiatry and Psychotherapy, University Medical Center Göttingen (UMG), Göttingen, Germany; 12 Center for Behavioral Brain Sciences (CBBS), Magdeburg, Germany; 13 German Center for Neurodegenerative Diseases, Göttingen, Germany; 14 Department of Nuclear Medicine, University Hospital Leipzig, Leipzig, Germany; 15 Neurosciences & Signaling Group, Department of Medical Sciences, Institute of Biomedicine (iBiMED), University of Aveiro, Aveiro, Portugal

**Keywords:** Neurology, Neuroscience

## Abstract

The role of physical fitness in cognitive reserve and brain maintenance remains unclear. We investigated the associations of muscular and aerobic fitness (VO_2_max) with markers of brain health and cognition in 353 cognitively unimpaired older adults (mean age = 72.77 ±  7.95 years; 177 females). Muscular fitness comprised handgrip strength, appendicular skeletal muscle mass, and Timed-Up-and-Go performance. Blood biomarkers of Alzheimer’s pathology (Aβ_1–42_/Aβ_1–40_, p-tau_217_, GFAP) and neuroplasticity (BDNF, VEGF, Cathepsin-B), gray matter (GM) volume and thickness, medial temporal lobe (MTL) tau burden ([¹⁸F]PI-2620 PET), white matter hyperintensities, and perivascular spaces (PVS) were assessed. Higher muscular capacity was associated with lower basal ganglia PVS volume, whereas aerobic fitness was related to greater MTL thickness and GM volume. Although fitness did not moderate pathology–cognition associations, VO₂max explained additional variance in verbal memory beyond tau. These findings indicate distinct associations of fitness with brain health, while providing limited support for cognitive reserve. Trial Registration: The study was registered in the German Clinical Trials Register (DRKS00032449; date of registration: 2025-05-07; recruiting ongoing).

## Introduction

Aging constitutes a multidimensional process associated with gradual cognitive decline and increased susceptibility to metabolic, cardiovascular, cerebrovascular, and neurodegenerative diseases, including Alzheimer’s disease (AD)^1–6^. Yet, the rate and magnitude of cognitive decline vary widely^1,2,7^, with some individuals maintaining high cognitive performance despite age or disease^1,3,8,9^. Understanding the mechanisms underlying such resilience has therefore become a major focus of aging research^10,11^. Among the factors proposed to support resilience, physical activity and structured exercise have emerged as promising contributors to preserved cognition, neuroplasticity, and brain health in older adults^12–14^. These effects may operate through mechanisms related to cognitive reserve (CR) and brain maintenance (BM)^10,11,15^.

BM refers to the capacity to preserve cognitive function in older age by resisting the decline of neural resources and the onset of neuropathological alterations^10,11,15^. Typical age-related changes in the brain include the accumulation of AD-related proteins, white matter (WM) lesions, enlargement of perivascular spaces (ePVS), and atrophy of gray matter (GM)^11,16–19^. The medial temporal lobe (MTL) is especially vulnerable to age-related atrophy and early accumulation of tau tangles^20–22^. Although longitudinal studies best capture trajectories of BM^10,11,15^, cross-sectional designs also provide valuable insights into the associations between lifestyle and neuropathology. In this respect, physical activity has repeatedly been linked to structural brain health, such as preserved GM volume, GM microstructure, and WM integrity^14,23–27^. Yet, the relationship to age-related pathological changes, such as ePVS, a potential marker for impaired brain homeostasis^28–30^, remains unclear. Furthermore, previous studies have reported mixed findings regarding the association between physical activity and AD biomarkers, with several observing no association with Aβ pathology^31–34^, while others linked higher physical activity to lower levels and slower increases in plasma p-tau as well as slower temporal lobe tau accumulation measured with PET^35–38^. However, the association between MTL tau burden and objective physical fitness measures, such as aerobic fitness, remains largely underexplored.

While BM denotes resistance to pathological changes, CR denotes the brain’s capacity to cope with or compensate for age-related changes or neuropathological damage^10,11,15^. Frequently operationalized using socio-behavioral proxies such as education, CR is shaped by diverse life experiences and lifestyle factors^11,39,40^. Evidence supporting physical activity as a contributor to CR remains mixed, ranging from positive effects^41–44^ to null findings^45–47^. These inconsistencies potentially reflect differences in the pathology markers, the measurement of physical activity, study populations, and statistical approaches^10,11,40^.

Epidemiological studies have reported positive associations between sustained physical activity and cognitive performance in older adults as well as larger hippocampal volumes across the lifespan^12,14,48^, with some evidence highlighting effects in the anterior hippocampus^49–54^, particularly on the left side. Neuroplasticity-related factors, such as brain-derived neurotrophic factor (BDNF), vascular endothelial growth factor (VEGF), and Cathepsin B, have also been proposed as biological mediators linking physical fitness to brain health and cognition^55–58^. Exercise-induced myokines, such as Cathepsin B, may stimulate neurotrophic signaling and neurogenesis^59–62^. Peripheral levels of BDNF have been positively associated with physical activity, hippocampal volume, and cognitive performance in older adults^12,13,49,63,64^. However, evidence for Cathepsin B and VEGF as mediators of exercise-related cognitive benefits remains inconsistent^65–70^.

Similarly, brain structural integrity, particularly hippocampal volume, has been proposed to mediate the relationship between physical activity and cognitive performance^14,24,49,52,71^, although supporting evidence is limited and largely based on cross-sectional studies.

In summary, accumulating evidence links physical activity to cognitive performance and brain integrity, suggesting a role of physical activity in CR and BM. However, most cross-sectional studies rely on self-reported physical activity, which is prone to recall bias and may not accurately reflect actual activity levels^72^. Objective measures such as aerobic (VO₂max, maximal oxygen uptake) and muscular fitness (as the inverse of sarcopenia) provide standardized indices of physical fitness^73^. To capture muscular fitness, we employed an investigator-defined summary index integrating complementary measures of muscle strength (handgrip strength), muscle quantity (appendicular skeletal muscle mass, ASMM), and physical performance (Timed-up-and-go test, TUG), reflecting the multidimensional assessment of muscle function recommended by the *European Working Group on Sarcopenia in Older People* (EWGSOP2)^74^. Muscular and aerobic fitness have been previously associated with cognitive performance^13,14,24,74,75^, offering complementary perspectives on inter-individual variability in cognitive outcomes.

To address the aforementioned limitations, the present study implemented a comprehensive assessment, comprising objective physical fitness measures, deep neuropsychological phenotyping, blood-based biomarkers related to AD pathology, astroglia reactivity and plasticity, and imaging-based brain and pathology outcomes in a cognitively normal aging cohort^76^. Regional MTL tau burden was assessed using the second-generation tau tracer [18F]PI-2620 in a subgroup of our sample^77–80^. We addressed four key questions: (1) whether higher physical fitness - both aerobic and muscular - is associated with better cognitive performance; (2) whether physical fitness is linked to lower brain pathology, supporting the concept of BM; (3) whether physical fitness serves as a proxy for CR; and (4) whether GM volume or thickness- especially in the hippocampus and MTL - and neuroplasticity markers mediate the relationship between physical fitness and cognitive outcomes.

## Results

A total of 353 cognitively normal older participants (177 females) were included in the study based on available muscular capacity data (age: *M* = 72.77 years, SD = 7.95; years of education: *M* = 14.93 years, SD = 2.38). The Supplemental Material provides uncorrected raw values for the physical fitness measures in Table S1, cognitive outcomes and pathology markers in Table S2, as well as the brain measures and plasticity-related factors in Table S3 together with the corresponding sample sizes (see Methods for further details).

**Fig. 1:**
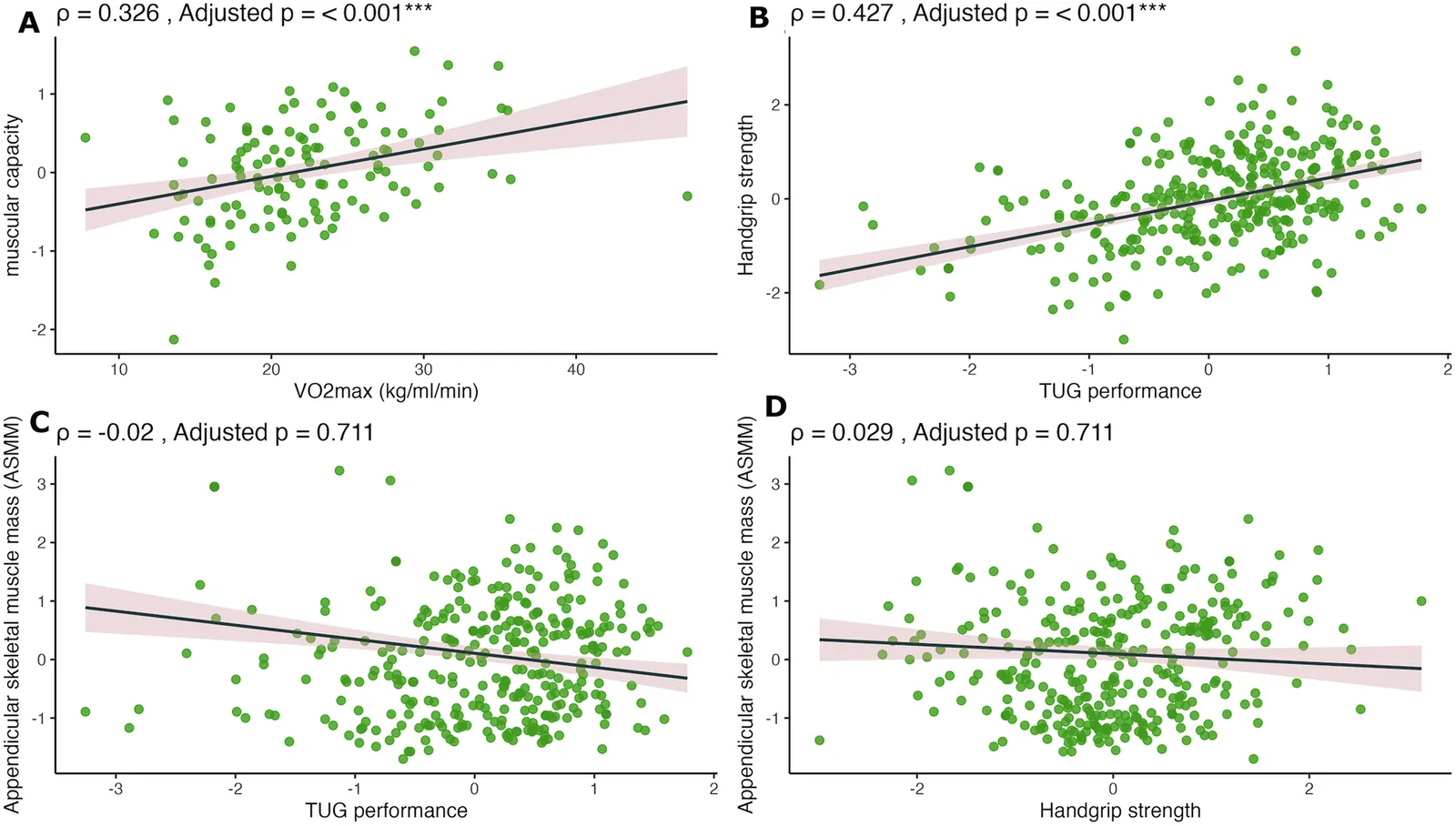
Bivariate associations between physical fitness outcomes. **A** shows VO_2_max (aerobic capacity, in ml/min/kg) and muscular capacity (z-composite: timed-up-and-go (TUG) test performance, maximal handgrip strength, and appendicular skeletal muscle mass [ASMM]). **B**–**D** show the muscular capacity outcomes separately. A linear trend line is shown with a 95% confidence interval (light pink area) to illustrate the relationship between the variables. *ρ*, Spearman’s rank correlation coefficient; Adjusted p: FDR-corrected (False Discovery Rate) *p*-value (Benjamini–Hochberg procedure).

Muscular capacity and VO₂max (available in a subsample of *N* = 140) were significantly positively correlated (*ρ* = 0.326, *p* < 0.001; Fig. 1), indicating that individuals with higher muscular capacity also exhibited higher aerobic capacity.

Within the muscular capacity composite, associations among its constituent measures, TUG performance (reverse coded for consistent interpretation), maximal handgrip strength, and ASMM as a proxy of muscle quantity, were evaluated (all z-standardized). Outliers were excluded from TUG (*n* = 4) and ASMM (*n* = 1). Better TUG performance was positively correlated with maximal handgrip strength (*ρ* = 0.427, *p* < 0.001; Fig. 1). In contrast, neither TUG performance nor maximal handgrip strength was significantly correlated with ASMM (*p*-values > 0.05; see Fig. 1).

Hypothesis 1: Association between physical fitness and cognitive performance

**Fig. 2:**
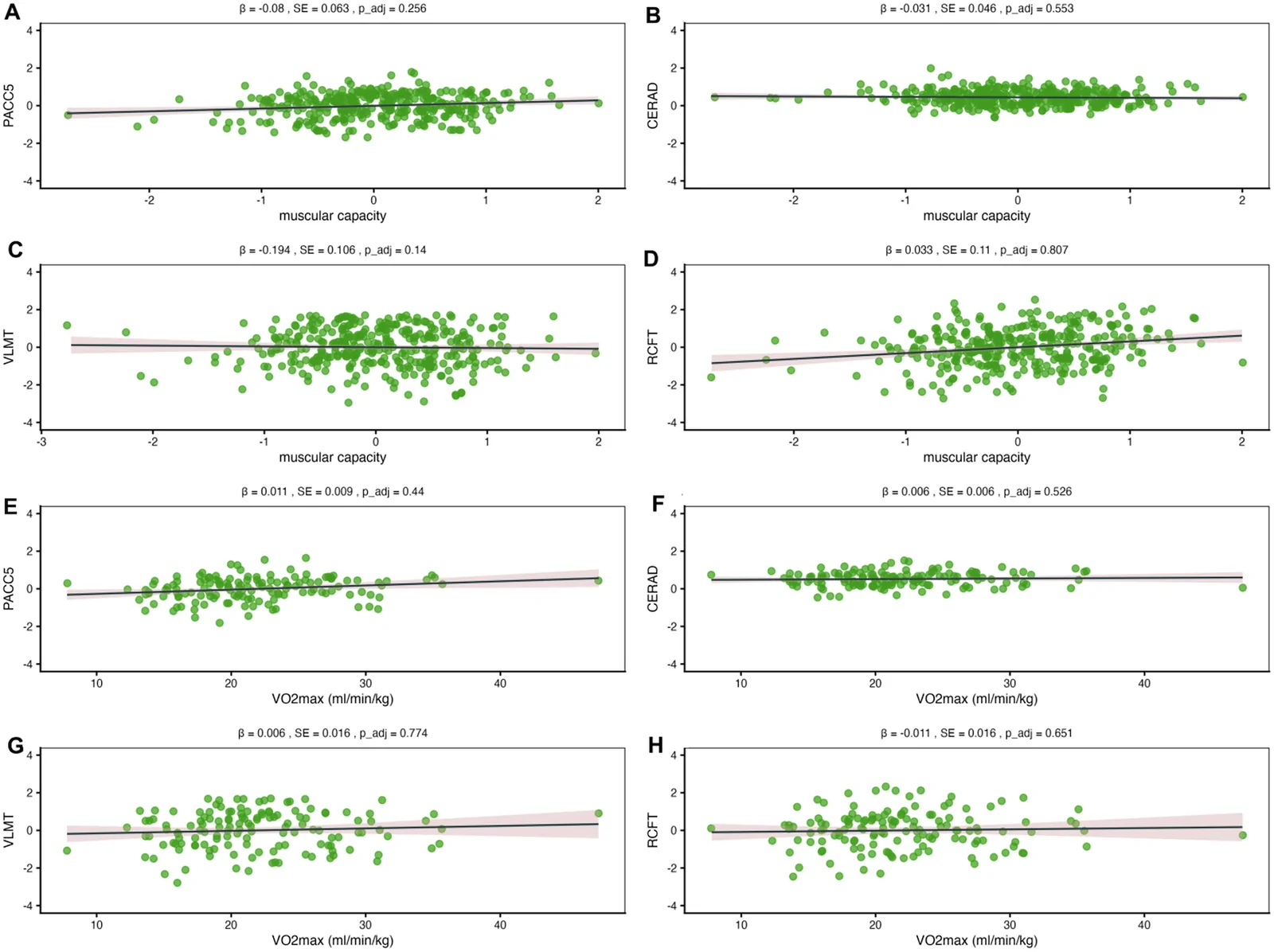
Linear regression plots of physical fitness and cognitive performance (raw *z*-score values). Physical fitness: muscular capacity (z-composite: TUG, maximal handgrip strength and ASMM; **A**–**D**) and VO₂max (aerobic capacity, in ml/min/kg; **E**–**H**); cognitive performance: CERAD, Consortium to Establish a Registry for Alzheimer’s Disease Plus version (z-composite); PACC5, Preclinical Alzheimer’s Cognitive Composite (z-composite); VLMT, German adaptation of Rey’s Auditory Verbal Learning Test (delayed verbal memory performance); ROCFT, Rey-Osterrieth Complex Figure Test (visuospatial delayed memory performance). Estimated regression lines are shown with a 95% confidence interval (light pink area). *β*, beta coefficient (slope); SE, standard error; adjusted p, FDR-corrected (False Discovery Rate) *p*-value (Benjamini-Hochberg procedure). All models included age, sex, and years of education as covariates.

To examine the hypothesis that higher physical fitness is related to better cognitive performance, separate regression models were specified for each cognitive outcome, adjusting for age, sex and years of education (see Fig. 2). These included late recall performance in the Verbal Learning and Memory Test (VLMT)^81^ and the Rey-Osterrieth Complex Figure Test (ROCFT)^82^ as well as the Preclinical Alzheimer’s Cognitive Composite (PACC5)^83^ and the Consortium to Establish a Registry for Alzheimer’s Disease-Plus (CERAD)^84^ score for global cognition (see “Methods”). No significant physical fitness by sex interaction was observed for either VO₂max or muscular capacity (all *p*-values > .05) on cognitive performance.

Muscular capacity was not significantly associated with global cognitive performance (PACC5: *β* = −0.080, SE = 0.063, *p*_adjusted_ = .256; CERAD: *β* = −0.031, SE = 0.046, *p*_adjusted_ = 0.553) or episodic memory performance (VLMT: *β* = −0.194, SE = 0.106, *p*_adjusted_ = 0.140; ROCFT: *β* = −0.033, SE = 0.110, *p*_adjusted_ = 0.807). Additional analyses examining the individual muscular capacity variables—TUG, ASMM, and maximal handgrip strength—also showed no significant associations with cognitive outcomes (Table S4). Similarly, VO₂max did not significantly predict global cognition (PACC5: *β* = 0.011, SE = 0.009, *p*_adjusted_ = 0.440; CERAD: *β* = 0.006, SE = 0.096, *p*_adjusted_ = 0.526) or episodic memory (VLMT: *β* = 0.006, SE = 0.016, *p*_adjusted_ = 0.774; ROCFT: *β* = −0.011, SE = 0.016, *p*_adjusted_ = 0.651). In summary, hypothesis 1 was not supported, as no significant associations were observed between physical fitness and cognitive performance.

Hypothesis 2: Physical fitness and resistance to brain pathology (Brain maintenance)

Next, we examined whether higher physical fitness was associated with lower age-related pathology, including basal ganglia (BG) and centrum semiovale (CSO) PVS volumes, white matter hyperintensities (WMH), and AD-related biomarkers (MTL tau PET DVR, p-tau_217_, Aβ₁₋₄₂/Aβ₁₋₄₀, and GFAP). For both physical fitness samples (muscular capacity: *N* = 353; aerobic capacity subsample: *N* = 140), outliers were excluded from p-tau_217_ (muscular capacity: *n* = 4; aerobic capacity: *n* = 1) and Aβ₁₋₄₂/Aβ₁₋₄₀ (muscular capacity: *n* = 1). To achieve normal distributions, plasma biomarker values were log-transformed before outlier removal.

**Fig. 3:**
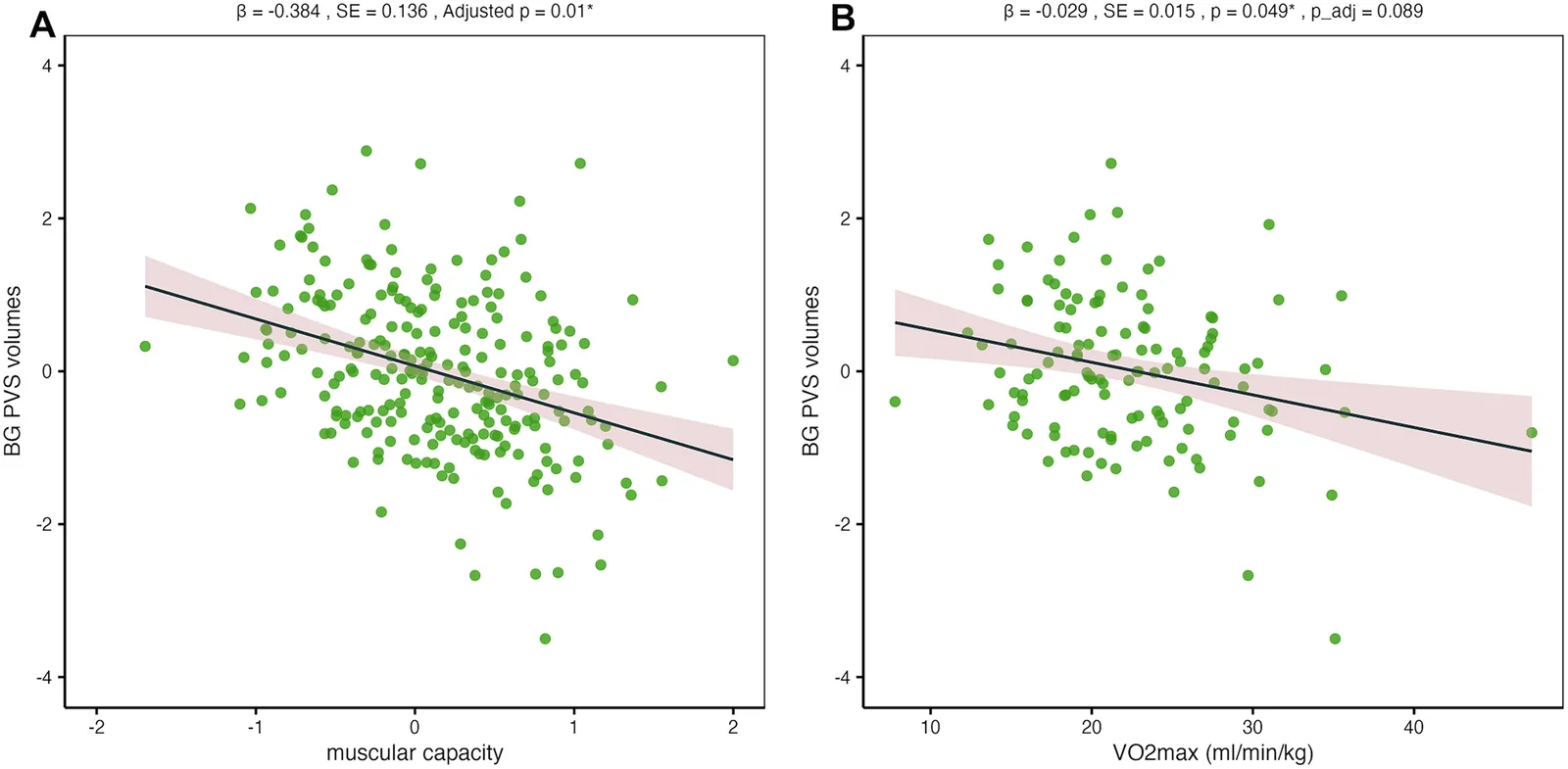
Association between physical fitness and pathology markers. **A** Effect of muscular capacity (z-standardized composite of TUG, ASMM, and maximal handgrip strength) on BG PVS volumes (basal ganglia perivascular spaces, normalized for BG volume). **B** Effect of VO₂max (aerobic capacity) on BG PVS volumes. The estimated regression line is displayed with its 95% confidence interval (light pink shaded area). *β*: regression slope (beta coefficient); SE: standard error; Adjusted *p*: FDR-corrected *p*-value (Benjamini–Hochberg procedure). *Indicates Adjusted *p* < 0.05. All models were adjusted for age and sex.

Higher muscular capacity was linked to lower BG PVS volumes (*β* = −0.384, SE = 0.136, *p*_adjusted_ = 0.010; Fig. 3A). For VO₂max, a negative association with BG PVS volumes was also observed (*p* = 0.049), but this did not survive False Discovery Rate (FDR) correction (*β* = −0.029, SE = 0.015, *p*_adjusted_ = 0.089; Fig. 3B). No significant associations were found between either physical fitness measure and the remaining pathology markers (all *p*_adjusted_ > 0.05). In supplementary analyses of the muscular capacity components, better TUG performance was associated with lower BG PVS volumes (*β* = −0.266, SE = 0.097, *p*_adjusted_ = 0.024; Fig. S1A), whereas no such association was observed for ASMM or maximal handgrip strength (Fig. S1B, C). Detailed associations between individual muscular capacity components and pathology markers are provided in the Supplemental Material (Fig. S1 and Table S5).

In summary, partial support for hypothesis 2 was observed: higher muscular capacity, in particular TUG performance, and aerobic capacity at a trend level, were associated with lower BG PVS volumes, whereas no significant associations were found for the remaining pathology markers assessed.

Hypothesis 3: Physical fitness as a proxy for cognitive reserve

First, associations between pathology markers and cognitive performance were evaluated. We then tested whether fitness moderated the association between pathology and cognition, consistent with the primary operationalization of CR^10^, and whether fitness explained additional variance in cognition beyond pathology.

In the muscular capacity sample, higher Aβ₁₋₄₂/Aβ₁₋₄₀ (i.e., less pathological levels) was associated with better global cognition (PACC5; *β* = 0.905, SE = 0.282, *p*_adjusted_ = 0.003), whereas higher GFAP levels were associated with poorer delayed recall on the VLMT (*β* = −0.444, SE = 0.190, *p*_adjusted_ = 0.037). No other pathology marker was associated with cognition (*p*_adjusted_ > 0.05). Muscular capacity did not moderate the association between Aβ₁₋₄₂/Aβ₁₋₄₀ and PACC5 performance (*β* = 8.07, SE = 5.36, *p*_adjusted_ = 0.133). Although a significant interaction was observed between GFAP and muscular capacity for VLMT performance (*β* = −0.57, SE = 0.28, *p*_adjusted_ = 0.044), the negative association between GFAP and memory was unexpectedly stronger in individuals with higher muscular capacity. However, this interaction was not observed in follow-up analyses of the individual muscular capacity components (all *p* > 0.05). Likewise, neither muscular capacity nor its individual components explained additional variance in cognition beyond pathology (all *p* > 0.05).

**Fig. 4:**
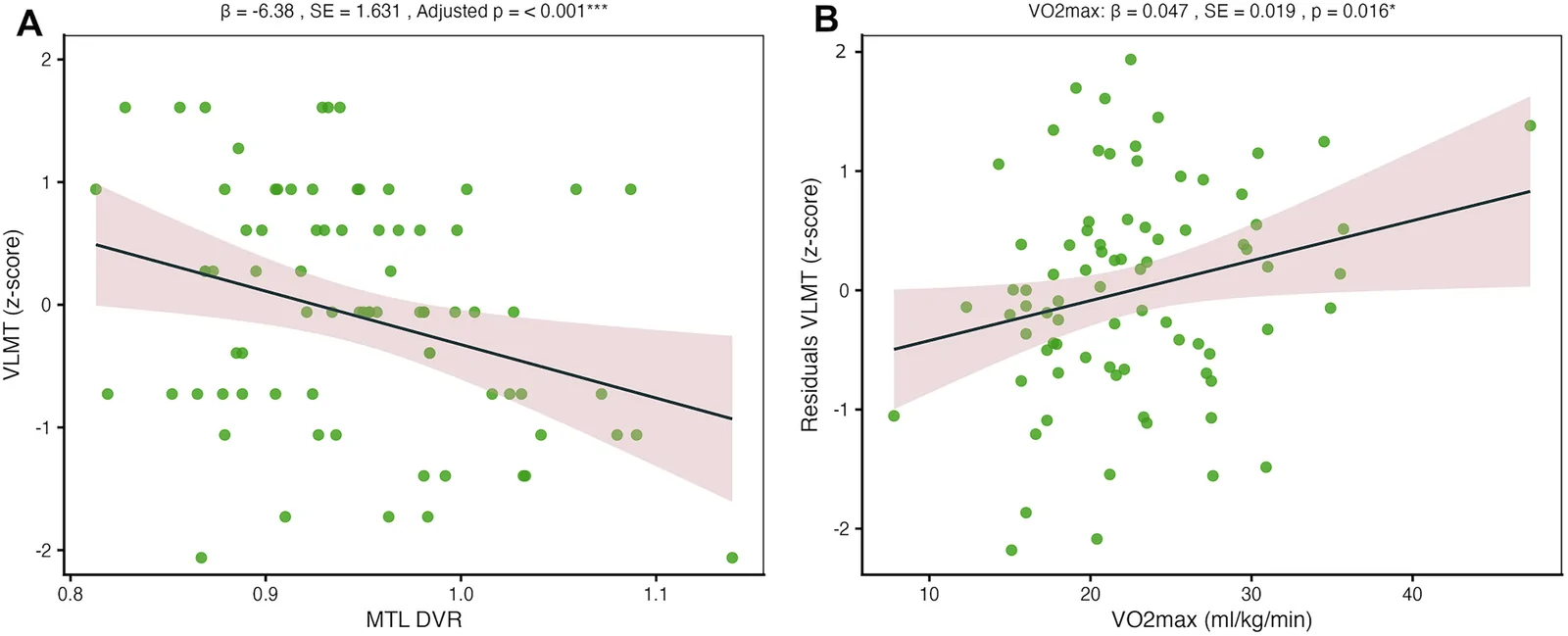
Associations of MTL tau burden and aerobic fitness with delayed verbal memory performance. **A** Association between medial temporal lobe (MTL) Tau PET DVR (predictor) and delayed verbal memory performance (VLMT *z*-score; dependent variable), adjusted for age and sex. **B** Association between VO₂max (aerobic capacity as predictor, ml/min/kg) and delayed verbal memory performance (VLMT, dependent variable) after removal of variance attributable to age, sex, and MTL Tau PET DVR. VLMT residuals were regressed on VO₂max to illustrate weak evidence for cognitive reserve. The estimated regression line is displayed with its 95% confidence interval (light pink shaded area). *β*: regression slope (beta coefficient); SE standard error; *Indicates *p* < 0.05.

Within the VO₂max subsample, higher MTL tau PET DVR was associated with poorer delayed recall on the VLMT (*β* = −6.38, SE = 1.63, *p*_adjusted_ < 0.001; Fig. 4A). No significant associations were observed between pathology markers and the other cognitive measures (all *p*_adjusted_ > 0.05). VO₂max did not moderate the association between MTL tau PET DVR and delayed verbal recall (*β* = −0.10, SE = 0.20, *p*_adjusted_ = 0.603). However, in a subsequent model including both VO₂max and MTL tau PET DVR, higher VO₂max was independently associated with better delayed verbal recall (*β* = 0.047, SE = 0.019, *p* = 0.016; Fig. 4B). Model comparison indicated that adding VO₂max significantly improved model fit (F(1,74) = 6.03, *p* = 0.016), explaining an additional 7.6% of the variance in delayed verbal recall beyond MTL tau PET DVR.

Taken together, VO₂max independently predicted better VLMT performance beyond MTL tau pathology but did not moderate the association between pathology and cognition. No evidence was found that either fitness measure acted as a CR proxy based on the primary moderation analysis.

Hypothesis 4: Neurobiological mechanisms linking physical fitness and cognitive function

Lastly, we examined whether brain structural and neuroplasticity-related markers represent potential pathways linking higher physical fitness to better cognitive performance. Potential mediators included hippocampal volumes (bilateral, left, and right for the anterior (aHCV), posterior (pHCV), and whole (wHCV) hippocampus), total GM volume, MTL thickness, and the neuroplasticity-related markers (BDNF, VEGF, and Cathepsin B). As neither aerobic fitness nor muscular capacity was associated with cognitive performance (all *p* > 0.05; see Hypothesis 1, Fig. 2), mediation analyses were not performed under our prespecfied analysis strategy (see Methods) was not significant.

The association between fitness and brain structure and plasticity markers (path a in mediation model) was examined separately for each physical fitness modality.

In the muscular capacity sample, higher muscular capacity was associated with lower right aHCV (β = −80.32, SE = 33.29, *p*_adjusted_ = 0.033), whereas no significant associations were observed for any other brain structural or plasticity marker. Supplementary analyses of the individual muscular capacity components (entered jointly in a single model per outcome; Tables S6–S8) showed that higher ASMM was associated with lower right aHCV (Table S6) and lower total GM volume (Table S7), whereas better TUG performance was associated with greater total GM volume and MTL thickness (Table S7).

**Fig. 5:**
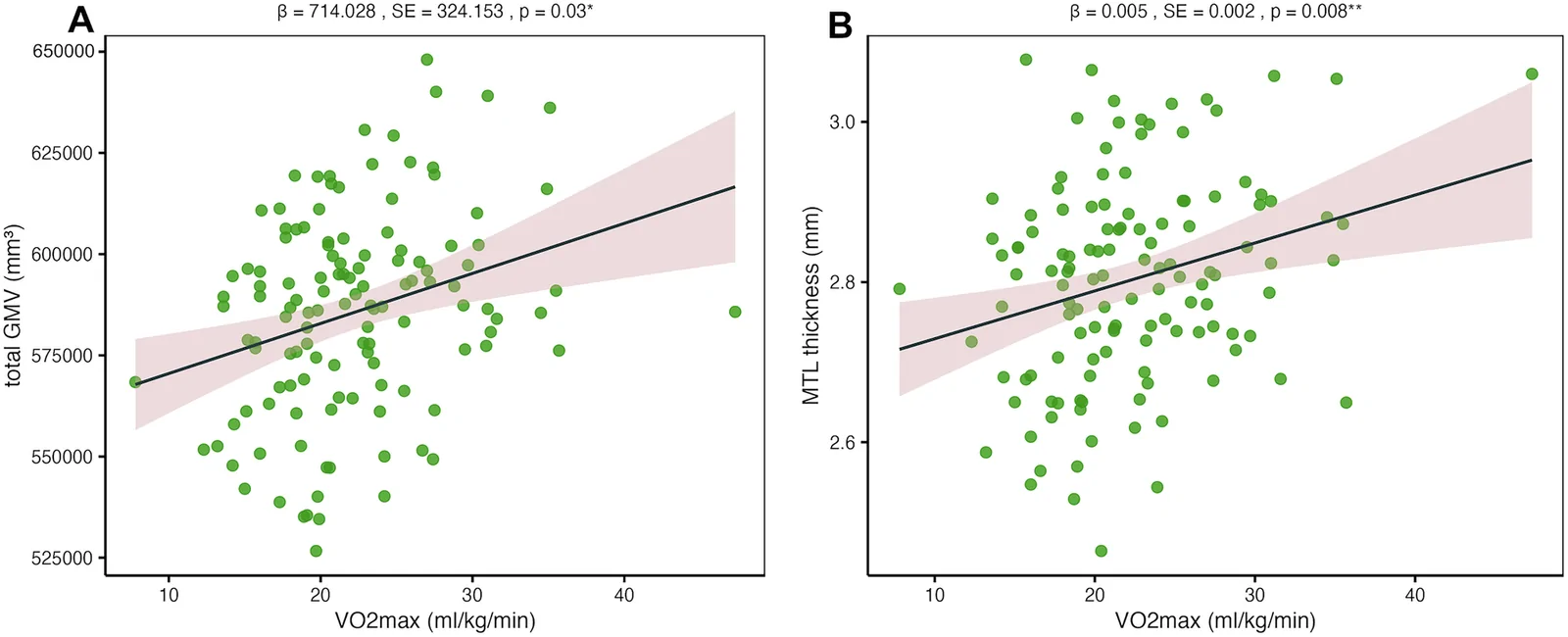
Association of aerobic fitness and brain structure. **A** Effect of VO_2_max (aerobic capacity as predictor, ml/min/kg) on total gray matter volume (GMV, mm^3^) and **B** on Medial temporal lobe (MTL) thickness (in mm). The estimated regression line is displayed with its 95% confidence interval (light pink shaded area). *β*: regression slope (beta coefficient); SE: standard error; Adjusted *p*: FDR-corrected p-value (Benjamini–Hochberg procedure). *indicates Adjusted *p* < 0.05. All models were adjusted for age and sex (raw values are plotted).

In the VO₂max sample, higher aerobic fitness was associated with greater total GM volume (*β* = 714.03, SE = 324.15, *p* = 0.03; Fig. 5A) and greater MTL thickness (*β* = 0.005, SE = 0.002, *p* = 0.008; Fig. 5B), whereas no other significant relationships with brain structural and neuroplasticity-related markers were detected.

Finally, we explored whether hippocampal volume and plasticity-related factors were associated, but correlations were not significant in either fitness sample (all *p*_adjusted_ > 0.05).

In summary, higher aerobic fitness was associated with greater brain structural integrity, whereas muscular capacity was unexpectedly associated with lower brain volumes. These negative associations were primarily driven by ASMM (muscle mass), whereas better TUG performance showed the expected positive associations with brain structure.

## Discussion

This study examined the role of physical fitness, specifically aerobic capacity and muscular capacity (used inversely as a proxy for sarcopenia), with respect to cognitive performance, brain structure, and markers of AD and cerebrovascular pathology among cognitively normal older adults. While neither fitness measure was associated with cognitive performance, aerobic fitness was related to more favorable brain structural measures and muscular fitness to lower BG PVS burden. These findings are broadly consistent with brain maintenance, although the cross-sectional design precludes conclusions about structural preservation over time. In contrast, muscle mass showed unexpected negative associations with brain volume and GFAP-related memory performance. Evidence for CR was limited, as aerobic fitness predicted verbal memory beyond tau pathology but did not moderate the tau–memory association.

Contrary to our first hypothesis, neither aerobic nor muscular capacity was associated with cognitive performance (in models not accounting for tau burden). Although a substantial body of animal research demonstrates that exercise promotes neurogenesis, synaptic plasticity, and learning and memory^57,58^, evidence linking physical fitness to cognitive performance in humans has been more heterogeneous. While several observational studies have reported positive associations, especially for episodic memory and executive function, with higher fitness predicting less future decline, these effects have been reported to be small and influenced by methodological differences, participant characteristics, and publication bias^85–87^. Our findings therefore add to the mixed human literature and suggest that, in cognitively normal older adults, higher physical fitness may be more closely related to brain structural integrity and lower cerebrovascular burden than to cognitive performance in a cross-sectional setting.

To the best of our knowledge, this is the first study to show that higher muscular capacity is associated with lower PVS volume in the BG. We observed a similar, although weaker, association for aerobic fitness in a smaller subsample. While PVS enlargement is likely multifactorial, chronic enlargement is widely recognized as pathological and has been found to be associated with cognitive deficits^88–91^. Although no human studies have so far directly examined the effects of aerobic exercise on PVS, it is plausible that aerobic activity may mitigate age-, inflammation-, and cardiovascular-related alterations by improving cerebrovascular and associated functions and, more broadly, supporting brain health^92–94^. Moreover, in a follow-up analysis of the individual measures of the muscular capacity composite, only poorer physical performance (TUG) was linked to increased BG PVS volumes. TUG performance, a marker of sarcopenia, has been associated with cognitive decline^74,75^, suggesting that muscular fitness warrants further investigation as a potential contributor to BM or resistance against cerebrovascular pathology. However, because of the cross-sectional design, reverse causality cannot be excluded. It is also plausible that subclinical cerebrovascular disease or early neurodegenerative processes contribute to reduced mobility and muscular performance. In addition, shared vascular or lifestyle-related risk factors may contribute to both reduced physical fitness and greater PVS burden. Longitudinal and interventional studies are therefore required to determine the temporal direction of these associations and to investigate potential mechanisms, including improved glymphatic clearance and preserved cerebrovascular integrity.

Moreover, we found no significant associations between objective measures of aerobic or muscular fitness and plasma markers of AD pathology (p-tau_217_, Aβ₁₋₄₂/Aβ₁₋₄₀) or GFAP. Similarly, a recent analysis from the population-based Rotterdam Study reported no association between accelerometer-measured physical activity and the same plasma AD biomarkers or cortical Aβ PET seven years later^32^. In contrast, a multicenter study in older adults with memory complaints found that self-reported moderate-to-vigorous physical activity predicted slower longitudinal increases in p-tau_181_, despite no association with baseline levels^36^. Likewise, evidence linking physical activity to GFAP, a marker of astrocyte activation associated with early Aβ pathology^95^, remains limited and inconsistent. While some human studies reported lower GFAP concentrations in more physically active older adults^96^, suggestive of reduced astrocytic inflammation and degeneration, others^97^, including the present study, found no association. Overall, evidence relating physical fitness to AD biomarkers remains mixed^36,98–102^, likely reflecting differences in fitness assessment, biomarker modality, participant characteristics (e.g., exclusion criteria, cognitive status), and statistical adjustment (e.g., for age and education).

Evidence that greater aerobic fitness contributes to CR against tau-related memory impairment was also limited. We found a negative association of MTL tau PET burden with memory, confirming previous studies^80,103,104^. Aerobic fitness did, however, not moderate the association between MTL tau burden and verbal memory. Recent data in non demented older adults showed that physical activity may attenuate the negative association between CSF tau and executive function (but not memory)^44^. Together, these findings suggest that the role of physical fitness in cognitive resilience to tau pathology remains uncertain^41–44,103^ and should be investigated in longitudinal studies including individuals across a broader spectrum of AD pathology.

Exercise is thought to promote cognitive health through molecular pathways involved in neuroplasticity. In animal models, exercise promotes hippocampal neurogenesis, synaptic plasticity, and memory through molecular pathways involving BDNF, peripheral myokines such as irisin and cathepsin B, and VEGF-mediated neurovascular adaptations^55–57,59–62,66^. However, translating these mechanisms to humans remains challenging. While some cross-sectional studies have identified hippocampal volume or plasticity-related markers as mediators of the association between aerobic fitness and cognition^52,54,105^, others have not^24,49,51,70^. In the present cohort, physical fitness was not associated with plasticity-related markers and, except for the association between aerobic fitness and memory after accounting for tau pathology, was not associated with cognition. These null findings may reflect the homogeneous, cognitively unimpaired sample, which limited variability in cognitive performance, and the small neuroplasticity marker subsample. Nevertheless, higher aerobic fitness was associated with greater MTL thickness and total GM volume, suggesting that fitness-related effects may be more readily detected using broader structural measures. Recent longitudinal diffusion MRI studies showed that exercise-induced improvements in fitness are more strongly associated with hippocampal microstructural changes than with hippocampal volume^26,27^, highlighting microstructural integrity as a potentially more sensitive marker of exercise-related brain adaptations.

Finally, there were also unexpected findings. Higher muscular capacity was associated with lower brain volume (adjusted for head size), contrary to our hypothesis. However, these associations were driven by ASMM, whereas TUG showed significant associations in the expected direction and handgrip strength showed no comparable relationships. Moreover, ASMM was not correlated with either TUG or handgrip strength, suggesting that muscle mass captures a distinct aspect of muscular capacity. Notably, ASMM was also correlated positively with BMI, indicating that it may partly reflect overall body size or body composition rather than functional muscular fitness in this cohort. Consistent with this interpretation, in our previous analysis of the same cohort^76^, higher ASMM loaded on a lifestyle component (based on principal component analysis) characterized by higher BMI and greater alcohol consumption. Likewise, higher muscular capacity was associated with a stronger, rather than weaker, negative association between GFAP and delayed memory, contrary to the hypothesized CR effect. As these findings were isolated, driven by muscle mass only, and not consistently observed across the remaining muscular fitness measures or pathology markers, they should be interpreted with caution and require replication in independent, longitudinal cohorts.

Our study has several strengths and limitations. The use of objective measures of aerobic and muscular fitness represents a methodological strength compared with studies relying on self-reported physical activity. However, the cross-sectional design precludes causal inference and limits the evaluation of BM. Longitudinal and interventional studies are now needed to determine whether different exercise modalities can reduce BG PVS burden. Further limitations should be noted. The sample consisted of cognitively normal, highly educated, community-dwelling Caucasian German individuals free of major neurological and systemic diseases. While these inclusion criteria were appropriate for investigating normal cognitive aging, the subgroup undergoing maximal cardiopulmonary exercise testing (VO₂max) was additionally required to meet exercise safety criteria, resulting in an even healthier subgroup for these analyses. Consequently, the restricted variability in fitness, cognition, and pathology measures may have reduced sensitivity to detect subtle associations, particularly in analyses involving VO₂max, and may therefore partly explain some of the null findings. These characteristics also limit the generalizability of our findings. In addition, several other measures, including tau PET and circulating neuroplasticity markers, were available only in subsamples, resulting in reduced statistical power for some analyses. Consequently, null findings, particularly for interaction analyses, should be interpreted with caution. Finally, physical fitness was assessed at a single time point and therefore may not reflect temporal variability or cumulative lifetime fitness, which may be more relevant to brain aging. Other domains of physical fitness, such as balance, flexibility, and functional fitness, were not assessed but may also contribute to cognitive health.

In conclusion, our findings suggest that physical fitness is associated with better brain health in old age, consistent with BM; however, evidence for direct associations with cognitive performance or cognitive reserve was limited. Future longitudinal and interventional studies incorporating multimodal neuroimaging and fluid biomarkers are needed to determine whether improving physical fitness promotes BM, enhances resilience to age-related pathology, or both.

## Methods

This study analyzed baseline data from an ongoing cohort investigating cognitively normal older adults (for details see ref. ^76^). The cohort is part of the central project *“Z03 – Human Molecular Imaging of Aging and SuperAging”* within the Collaborative Research Centre 1436 (www.sfb1436.de), *“Neural Resources of Cognition”*. Its aim is to characterize factors or mechanisms related to successful brain and cognitive aging in deeply phenotyped older individuals using a combination of cross-sectional and longitudinal assessments. Participants undergo a comprehensive evaluation including neuropsychological testing, structural and functional neuroimaging, tau PET, molecular and genetic biomarker analyses, physical fitness assessments, and lifestyle questionnaires. For the present study, only baseline data were used, with a focus on the fitness data.

### Participants

The sample comprised 353 cognitively normal older adults with available muscular capacity data (177 females), aged 60–94 years (*M* = 72.77, SD = 7.95), all independently living and with relatively high educational attainment (*M* = 14.93 years, SD = 2.38).

General inclusion and exclusion criteria for this study cohort have been described in detail previously (see ref.^76^). Briefly, general eligibility required the absence of objective cognitive decline (according to age-, sex-, and education-adjusted norms of the CERAD-Plus Neuropsychological Assessment Battery), no neurological or psychiatric conditions, lack of major medical illness, no diagnosis of diabetes, no substance-related addiction, and no intake of medications affecting cognition.

Beyond these general eligibility criteria, additional safety screening was applied to the physical fitness assessments. For maximal cardiopulmonary exercise testing (spiroergometry), participants were excluded only if they had severe, acute, or uncontrolled medical conditions that could increase the risk associated with maximal exertion. These included severe cardiovascular disease (e.g., acute coronary syndrome, symptomatic severe aortic stenosis, congestive heart failure, or resting blood pressure >180/100 mmHg), severe neuromuscular disorders (e.g., active myositis), or severe metabolic/endocrine conditions (e.g., obesity; BMI > 30 kg/m²).

Importantly, these criteria applied only to the spiroergometry protocol. Participants who were not eligible for maximal exercise testing but otherwise met the study inclusion criteria completed a shorter fitness assessment (for muscular capacity assessment). Likewise, temporary conditions or injuries (e.g., orthopedic injuries or acute infections) did not result in permanent exclusion; instead, fitness testing was postponed until full recovery.

The study protocol was approved by the Ethics Committee of the Medical Faculty at Otto-von-Guericke University Magdeburg (approval no. 200/19) and conducted in accordance with the Declaration of Helsinki. All participants provided written informed consent and received financial compensation. The study was registered with the German Clinical Trials Register (DRKS00032449).

### Experimental design

**Fig. 6:**
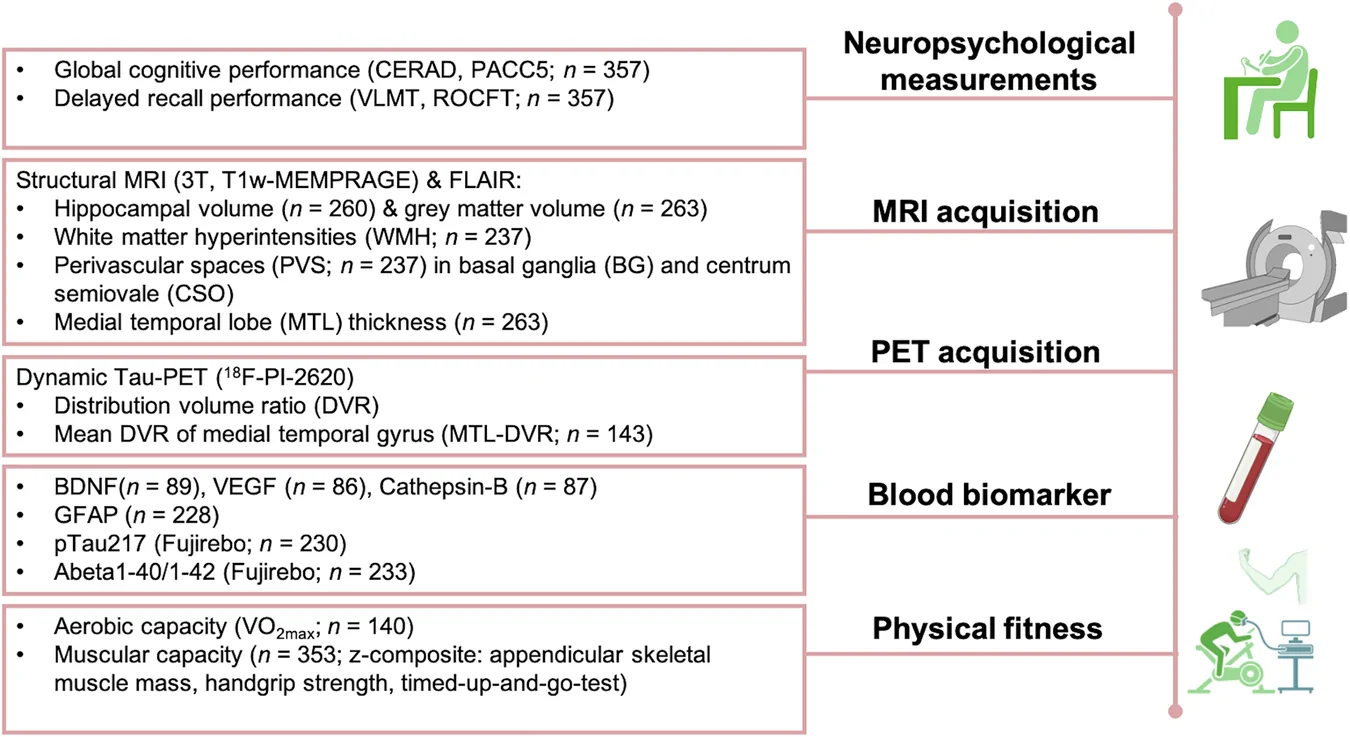
Overview of study methodology and schematic presentation of the primary variables and measures. CERAD, Consortium to establish a Registry for Alzheimer’s Disease (z-composite using CERAD-Plus battery); PACC5, preclinical Alzheimer’s composite (z-composite); VLMT, German adaptation of Rey’s Auditory Verbal Learning Test (delayed verbal memory performance); ROCFT, Rey-Osterrieth Complex Figure Test (visuospatial delayed memory performance); BG PVS, basal ganglia perivascular spaces; CSO PVS, centrum semiovale perivascular spaces; BDNF, brain derived neurotrophic factor; VEGF, vascular endothelial growth factor, GFAP, glial fibrillary acidic protein; VO_2_max, maximal oxygen consumption (ml/min/kg); ASMM, appendicular skeletal muscle mass (in kg); TUG, timed-up-and-go-test (in seconds).

All analyses were based on the baseline data (Fig. 6). The protocol included a multimodal assessment comprising: comprehensive neuropsychological testing, high-resolution MRI, [^18^F]PI-2620 tau PET imaging (subsample of participants), venous blood collection for molecular and genetic markers, and objective measures of aerobic and muscular fitness. Testing sessions were distributed to optimize data quality and reduce participant fatigue, ensuring reliable measures across all modalities. The number of available data for each measurement modality is also provided in Fig. 6 and Tables S1–S3.

### Neuropsychological measurements

Neuropsychological testing was conducted across two separate sessions. During the initial session, participants (*n* = 357) underwent the CERAD-Plus to confirm normal cognitive performance. Raw scores were converted to age-, sex-, and education-adjusted z-scores. Participants were excluded if scores indicated evidence of possible cognitive limitations based on the neuropsychological Jak/Bondi criteria^76,106^ or MMSE < 26 (for details see ref. ^76^).

The second testing session occurred approximately one week later and consisted of an extended cognitive battery covering a broader range of domains. Episodic memory was probed using the VLMT^81^, the German analog of the Rey Auditory Verbal Learning Test. Additional memory assessments included the Logical Memory II subtest from the Wechsler Memory Scale (WMS^107^) and the Free and Cued Selective Reminding Test (FCSRT^108^). Visuospatial and constructional abilities were examined using ROCFT^82^. Attention and processing speed were evaluated using the Symbol Digit Modalities Test (SDMT^109^). Verbal fluency was further assessed using both semantic and phonemic fluency tasks, including task-switching components, based on the Regensburger Wortflüssigkeitstest (RWT)^110^. A composite z-score was computed following the PACC5 framework^84^, derived from the mean of MMSE, delayed logical memory (WMS-R), SDMT, FCSRT (free and total recall), and RWT category fluency tasks.

### MRI acquisition and processing

High-resolution structural MRI was performed on a 3T scanner (MAGNETOM Skyra, Siemens) using a multi-echo magnetization-prepared rapid acquisition gradient-echo (MEMPRAGE) sequence. Images were acquired with an isotropic voxel size of 0.8 mm, echo times (TEs) of 1.85, 3.75, 5.65, and 7.55 ms, a repetition time (TR) of 2560 ms, an inversion time (TI) of 1100 ms, and a flip angle of 7°. The acquisition time was approximately 6.5 min. All subsequent volumetric analyses were performed on the echo-combined T1-weighted images. In addition, a fluid-attenuated inversion recovery (FLAIR) sequence was acquired with 1.0 mm isotropic resolution (TR = 5 s, TE = 393 ms, TI = 1.8 s), resulting in an acquisition time of 4 min 37 s.

Quantification of hippocampal subfields was performed using the Automatic Segmentation of Hippocampal Subfields (ASHS) algorithm. T1-weighted scans (*n* = 260) were resampled to a voxel resolution of 0.8 × 0.4 × 0.4 mm, and segmentation was guided by the 3 T Pennsylvania Memory Center atlas^111^. This process enabled extraction of volumetric measures for the aHCV, pHCV, and total hippocampal volume (wHCV). The segmentation protocol adhered to the Harmonized Protocol on T1-weighted images^111,112^, supplemented by inclusion of the alveus and fimbria regions^113^.

Each segmented image was subjected to independent visual inspection by two blinded raters to ensure anatomical accuracy. Manual corrections were applied in 62 hemispheres, addressing systematic segmentation errors such as underestimation of the lateral hippocampal boundary or misclassification of the choroid plexus^111^. Adjustments followed guidelines from the ASHS-T2 and Harmonized Protocols^111^. Following visual quality control and manual refinement, volumetric data for aHCV, pHCV, and wHCV were extracted for each hemisphere and aggregated to generate bilateral measures for subsequent analyses.

To quantify volumetric brain parameters, high-resolution T1-weighted structural images (*n* = 263) were processed using FreeSurfer to extract total GM volume and via *samseg* to extract total intracranial volume (TIV)^76^.

To control for interindividual variability in cranial size, a linear regression-based normalization procedure was employed in accordance with established protocols^114^. Specifically, adjusted volumes (Voladj) were computed using the equation:

$${Voladj}={Volrawi}-{{b}}({TIVi}-{meanTIV})$$

Here, Volrawᵢ represents the unadjusted volume of a given region of interest (ROI) for subject *i*, *b* denotes the slope of the linear regression of raw volume on TIV, and meanTIV corresponds to the sample mean. These adjusted volumetric measures were subsequently used in statistical analyses.

PVS, indicative of cerebral small vessel disease if enlarged, were segmented within the CSO and BG using a validated semi-automated pipeline that integrated information from T1-weighted and FLAIR sequences (*n* = 237)^29,115^. The CSO ROI encompassed the supratentorial white matter excluding BG structures, while the BG ROI comprised the caudate, lentiform nucleus, thalamus, and adjacent internal and external capsules. Although these regions do not precisely correspond to anatomical boundaries, their delineation follows established conventions from visual rating protocols^116^. PVS burden was quantified as fractional volume (PVS_volume/ROI_volume) and normalized using the *bestNormalize* algorithm. This procedure identified a logarithmic transformation as the optimal solution for both CSO and BG PVS to reduce distributional skewness and improve the approximation to normality.

WMH were segmented from T1-weighted and FLAIR images using the AI-enhanced Lesion Segmentation Toolbox (LST-AI), incorporating deep learning-based lesion detection (*n* = 237)^117^. Total and regional WMH volumes (cortical lobes, corpus callosum, and internal/external capsules) were quantified based on the USCLobes Atlas^118^. To address non-normality, WMH volumes underwent a Box-Cox transformation, identified by the *bestNormalize* procedure^119^ in R as the optimal transformation for this variable. TIV correction was performed using linear regression, and residuals from the TIV-WMH model were used for subsequent analyses.

MTL thickness was estimated from the T1-weighted images using FreeSurfer (*n* = 263). Bilateral MTL thickness (mm) was calculated as the average across both hemispheres, including entorhinal, parahippocampal, and fusiform cortical thickness. Although the fusiform gyrus is classically considered part of the inferior temporal cortex, the anterior-to-middle portion of the FreeSurfer fusiform label overlaps with Brodmann area 36 (perirhinal cortex) and was therefore included in the composite. Thus, the composite primarily reflects medial temporal cortical integrity while incorporating part of the inferior temporal cortex.

### PET acquisition and analysis

Regional tau burden was measured with the second-generation tau tracer [18F]PI-2620, selected for its high specificity to tau isoforms relevant to AD and reduced off-target binding compared to earlier tracers^77,78,120^. Simultaneous PET-MRI acquisition was conducted using the Siemens Biograph mMR system at the DZNE in Magdeburg. Participants (*n* = 143; available scans included until 02/2025) received an intravenous bolus injection of a single average tracer dose of 183 MBq (SD = 1.7 MBq)^77,80^. Dynamic PET data were acquired over 60 min and reconstructed into 34 frames^80^.

Motion correction and co-registration of PET frames to individual T1-weighted MRIs were performed. T1-weighted images acquired during the MR-PET scan were segmented with FreeSurfer to derive regions of interest. DVR maps were computed using the Multilinear Reference Tissue Model 2 (MRTM2) implemented in *QModeling* (Matlab R2024V)^121,122^. The globus pallidus and inferior cerebellum were designated as high-binding and reference regions, respectively^77,79,80^. Mean DVR values were extracted for key MTL structures, including the entorhinal cortex, parahippocampal gyrus, fusiform gyrus, and amygdala. We did not include the hippocampus in our MTL ROI due to off-target binding in the choroid plexus, as we did not apply partial volume correction.

### Blood-based biomarkers

On the same day as the physical fitness assessment (see below), venous blood was collected under standardized fasting and substance-abstention conditions (2 h for food, 4 h for nicotine/caffeine). Serum samples were cooled, centrifuged (15 min, 4 °C, 1100 × *g*), aliquoted, and stored at −80 °C. Target analytes were quantified via enzyme-linked immunosorbent assays (ELISA) in duplicate: BDNF (custom sandwich ELISA adapted from ref. ^123^; *n* = 89), VEGF (R&D Systems; *n* = 86), and Cathepsin B (Abcam; *n* = 87). Analyses adhered to quality guidelines from the FDA (2001) and EMA (2013).

On a separate occasion, EDTA plasma samples were obtained. Blood biomarkers for amyloid and tau pathology were analyzed at the Department of Psychiatry and Psychotherapy, University Medical Center Goettingen & DZNE, Goettingen. Aliquots of 500 μl EDTA–blood plasma stored at −80 °C in Matrix 0.5 mL tubes (Thermo Scientific) were thawed at room temperature, mixed vigorously for 5–10 s and centrifuged for 10 min at 10,000 × *g* at room temperature in a fixed-angle rotor. Plasma Aβ_1-40_, Aβ_1-42_ (*n* = 230 each), p-tau_217_ (*n* = 233), and GFAP (*n* = 228) concentrations were measured using commercially available immunoreaction cartridges on the fully automated Lumipulse G600II system (see ref. ^76^ for details).

### Assessment of physical fitness

Physical fitness assessment included measurements of both aerobic (VO_2_max) and muscular capacity (inverse proxy of sarcopenia). However, due to time constraints in the later stages of the study, only muscular capacity was assessed, resulting in a larger sample size for this measure (*n* = 353). Consequently, data on both muscular and aerobic capacity were available for only a subsample of participants (*N* = 140; comprehensive physical fitness assessment).

Muscular capacity was evaluated through a composite of maximal handgrip strength (SAEHAN dynamometer; average of three maximal voluntary contractions per hand), TUG test, and ASMM assessed via bioelectrical impedance analysis (BIA 101 BIVA PRO). A standardized z-score composite was derived from these measures, with TUG scores inverted (TUG x –1) to ensure consistent interpretation: higher scores reflected superior muscular function.

Cardiorespiratory fitness was quantified using a symptom-limited cardiopulmonary exercise test (CPET) on a cycle ergometer (Ergoselect 200, COSMED). The protocol commenced at 25 W, with incremental increases of 25 W every 3 min, flanked by warm-up and cool-down phases (0 W, 3 min each). Expired gases were continuously monitored via breath-by-breath analysis (Quark CPET), alongside heart rate (12-lead ECG), blood pressure, and subjective exertion (Borg Scale 6–20). Termination criteria included a VO₂ plateau, respiratory exchange ratio > 1.10, or volitional fatigue.

### Statistical analyses

All statistical analyses were performed using R (version 4.3.2) within the RStudio environment (version 2023.9.1.494). Data visualization was conducted utilizing the *ggplot2* package, as part of the *tidyverse* package^124^, and the *corrplo*t package^125^.

Prior to conducting inferential analyses, the assumptions underlying linear models were assessed. Normality of residuals was examined via the Shapiro–Wilk test, complemented by visual inspection of histograms and Q–Q plots. Variables that were right-skewed were log-transformed prior to analysis and outlier removal. Extreme outliers were defined as data points exceeding ±3 times the interquartile range and were excluded from subsequent analyses. Linearity was evaluated by plotting residuals against fitted values, and log transformation was applied in cases of substantial skewness. Homoscedasticity was assessed using the Breusch–Pagan test, and the Durbin–Watson test was employed to verify the independence of residuals. Multicollinearity was assessed using variance inflation factors (VIF).

An alpha threshold of *p* < 0.05 was used for all hypothesis testing. Correction for multiple comparisons was implemented using the Benjamini–Hochberg FDR procedure to minimize the likelihood of Type I errors in the context of multiple inferential tests.

Hypothesis 1: Association between physical fitness and cognitive performance

First, we investigated the hypothesis that higher physical fitness relates to better cognitive function. Linear regression models were fitted using the *lm* function from the stats package. Aerobic and muscular fitness were analyzed in separate models predicting cognitive performance across the CERAD, PACC5, VLMT, and ROCFT, adjusting for age, sex, and years of education. Physical fitness × sex interaction terms were initially included and removed if non-significant. FDR correction was applied across the four cognitive outcomes within each fitness subsample. In supplementary analyses, the primary models were repeated with TUG, ASMM, and maximal handgrip strength entered simultaneously as individual predictors instead of the muscular capacity composite.

Hypothesis 2: Physical fitness and resistance to brain pathology

Second, we investigated the hypothesis that higher physical fitness predicts less age-related pathology (i.e., resistance or BM). Linear regression models were fitted with muscular fitness (z-standardized composite score) or aerobic fitness (VO₂max) as predictors and fractional BG and CSO PVS volume, whole-brain WMH volume, MTL tau PET load (distribution volume ratio, DVR), plasma p-tau_217_, the Aβ₁₋₄₂/Aβ₁₋₄₀ ratio, and GFAP as outcomes. All models were adjusted for age and sex. FDR correction was applied across the seven pathology markers within each fitness subsample. In supplementary analyses, the primary models were repeated with TUG, ASMM, and maximal handgrip strength entered simultaneously as individual predictors instead of the muscular capacity composite.

Hypothesis 3: Physical fitness as a proxy for cognitive reserve

**Fig. 7:**
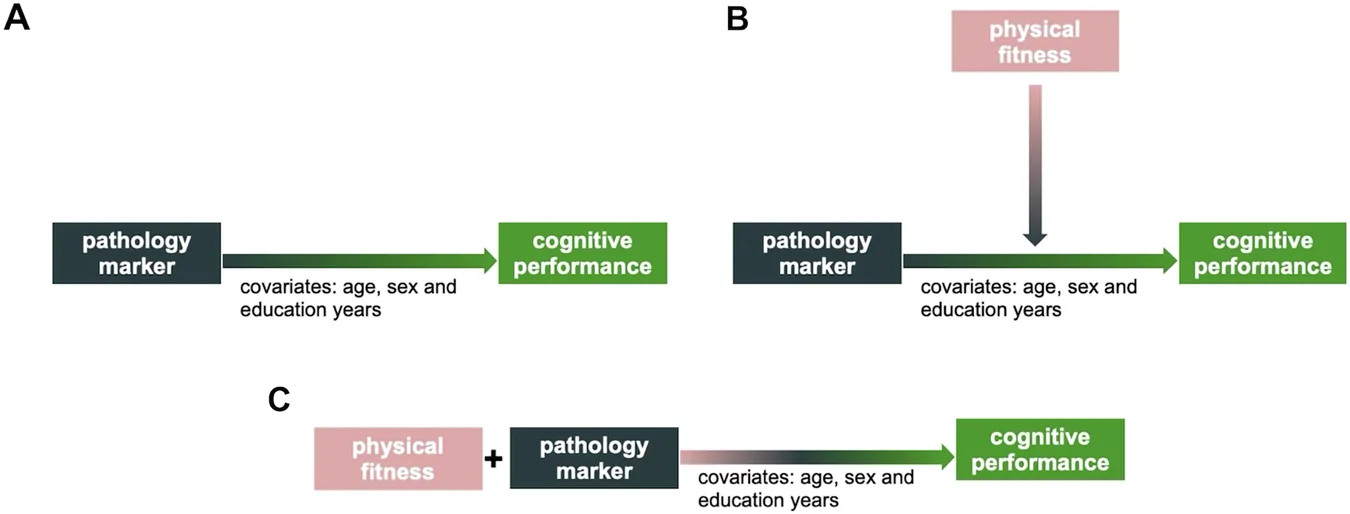
Cognitive reserve analysis. **A** Baseline-condition: influence of pathology markers on cognitive performance. **B** Moderation analysis: examining the interaction between physical fitness and pathology markers on cognitive outcomes. **C** Analysis for weak evidence of cognitive reserve: model including both physical fitness and pathology markers as predictors of cognitive performance. Cognitive performance measures: PACC5, CERAD, VLMT, ROCFT; pathology marker: BG PVS, CSO PVS, WMH, MTL DVR, p-tau_217_, Aβ₁₋₄₂/ Aβ₁₋₄₀, GFAP; physical fitness markers: VO2max and muscular capacity; covariates: age (in years) and sex.

To examine whether physical fitness serves as a proxy for CR, a multistep analytical strategy was applied. First, linear regression models assessed associations between neuropathological markers (BG PVS, CSO PVS, WMH, MTL DVR, p-tau_217_, Aβ₁₋₄₂/Aβ₁₋₄₀, and GFAP) and cognitive performance (Fig. 7A). CERAD and PACC5 were selected as global cognitive measures, whereas VLMT and ROCFT assessed episodic memory. Where a pathology marker was significantly associated with cognition, physical fitness (VO₂max or muscular capacity) × pathology interaction terms were added to test for moderation, consistent with the CR framework^10,11^(Fig. 7B).

If no significant interaction was observed but physical fitness independently predicted cognition, nested models with and without the fitness variable were compared using ANOVA to determine whether fitness improved model fit (Fig. 7C), representing a weaker form of CR evidence than moderation^10^. All models were adjusted for age, sex, and years of education. FDR correction was applied separately for each cognitive outcome across the seven pathology markers within each fitness subsample. Secondary analyses repeated these procedures using TUG, ASMM, and maximal handgrip strength instead of the muscular capacity composite.

Hypothesis 4: Neurobiological mechanisms linking physical fitness and cognitive function

**Fig. 8:**
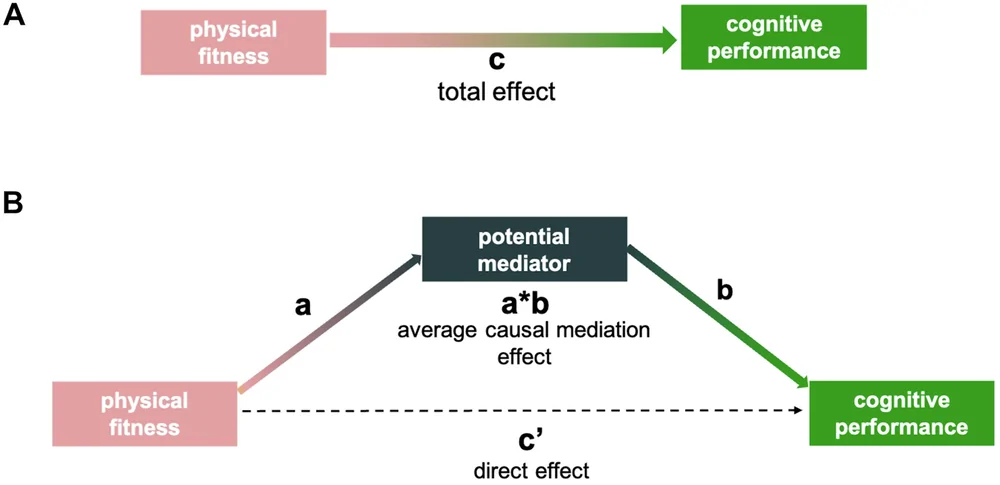
Planned mediation analysis examining the association between physical fitness and cognitive performance. **A** Total effect (c): the overall effect of physical fitness markers (VO_2_max and muscular capacity, separately) on cognitive performance measures (PACC5, CERAD, VLMT, ROCFT). **B** Mediation decomposition: the indirect effect (average causal mediation effect, a*b) represents the portion of the effect transmitted through the mediator, while the direct effect (c’) represents the remaining effect of physical fitness on cognition not explained by the mediator. The total effect is the sum of the direct and indirect effects, expressed as c = c’ + (a ∗ b).

To investigate potential neurobiological mechanisms linking physical fitness to cognition, mediation analyses were planned (Fig. 8). Models were estimated only when physical fitness (VO₂max or muscular capacity) was significantly associated with both the proposed mediators (brain structural measures or neurotrophic factors) and the cognitive outcomes. Analyses were to be conducted using the mediation package in R with nonparametric bootstrapping (50,000 iterations)^126^. When no significant total effect was observed, only associations between physical fitness and the proposed mediators (path a; Fig. 8) were examined.

All models were adjusted for age, sex, and years of education. TIV was not included because volumetric brain measures had been pre-adjusted during preprocessing. FDR correction for fitness associations was applied separately across (i) four cognitive outcomes, (ii) nine hippocampal volume measures, and (iii) three neuroplasticity markers (BDNF, VEGF, and cathepsin B) within each fitness subsample. MTL thickness and total GM volume were analyzed separately without multiple-comparison correction. Supplementary analyses repeated these models using TUG, ASMM, and maximal handgrip strength instead of the muscular capacity composite.

Associations between hippocampal volume (total, anterior, and posterior) and peripheral neurotrophic biomarkers (BDNF, VEGF, and Cathepsin B) were assessed using Pearson correlations with Benjamini–Hochberg FDR correction.

In addition, associations between hippocampal volume (total, anterior, and posterior) and peripheral neurotrophic biomarkers (BDNF, VEGF, and Cathepsin B) were explored using Pearson correlations with Benjamini–Hochberg FDR correction.

### Ethics approval and consent to participate

The study design and protocol were approved by the local Ethics Committee of the Medical Faculty, Otto-von-Guericke University Magdeburg (200/19) and were carried out in accordance with the code of ethics of the World Medical Association (Declaration of Helsinki, 1967). Written informed consent was obtained from all participants.

## Supplementary information

Supplemental Material

## Data Availability

The datasets generated and/or analyzed during the current study are not publicly available due to ethical and data protection regulations governing this cohort but are available from the corresponding author on reasonable request.

## Code availability

The underlying code for this study is not publicly available for proprietary reasons.
