# Supplementary material for: Associations of physical fitness with brain structure, pathology and cognition in cognitively normal older adults
Source: NPJ Aging. 2026 Sep 16;12(1):129. doi: 10.1038/s41514-026-00512-4 (PMC13582920; doi:10.1038/s41514-026-00512-4)
Supplement: Supplementary file 1 [file 41514_2026_512_MOESM1_ESM.docx]

Associations of physical fitness with brain structure, pathology and cognition in cognitively normal older adults

## Table S1.

|  | | | | | |
| --- | --- | --- | --- | --- | --- |
| **Outcome** | **N** | **M** | **SD** | **Median** | **IQR** |
| Handgrip | 353 | 30.87 | 9.50 | 29.83 | 14.75 |
| ASMM | 353 | 20.42 | 4.71 | 20.20 | 7.30 |
| TUG | 353 | 6.09 | 1.52 | 5.76 | 1.62 |
| VO2max | 140 | 22.18 | 5.92 | 21.30 | 7.52 |

Descriptive statistics of physical fitness parameters. Uncorrected Raw values are presented as mean (M), standard deviation (SD), median and interquartile range (IQR). Handgrip, maximal handgrip strength (average of three maximal voluntary contractions per hand); ASMM, appendicular skeletal muscle mass (in kg); TUG, timed-up-and-go-test (in seconds); VO2max, maximal oxygen consumption (in ml/min/kg).

## Table S2

| **Outcome** | **N** | **M** | **SD** | **Median** | **IQR** |
| --- | --- | --- | --- | --- | --- |
| Age | 357 | 72.77 | 7.95 | 71.00 | 15.00 |
| Education Years | 357 | 14.93 | 2.38 | 15.00 | 3.00 |
| PACC5 | 357 | −0.01 | 0.65 | 0.05 | 0.84 |
| VLMT | 357 | 10.33 | 2.96 | 10.00 | 5.00 |
| ROCFT | 357 | 17.95 | 6.10 | 18.00 | 8.00 |
| BG PVS | 237 | 0.17 | 0.12 | 0.14 | 0.11 |
| CSO PVS | 237 | 0.81 | 0.64 | 0.67 | 0.75 |
| WMH | 237 | 4.65 | 7.01 | 1.76 | 4.29 |
| MTL DVR | 143 | 0.93 | 0.06 | 0.93 | 0.09 |
| ptau 217 | 230 | 0.13 | 0.10 | 0.10 | 0.05 |
| Aβ42/40 | 233 | 0.09 | 0.01 | 0.09 | 0.01 |
| GFAP | 228 | 4.06 | 0.37 | 3.98 | 0.49 |

Descriptive statistics of demographic and pathology parameters. Uncorrected Raw values are presented as mean (M), standard deviation (SD), median and interquartile range (IQR). Age and Education years in years; CERAD, Consortium to establish a Registry for Alzheimer’s Disease Plus version (z-composite); PACC5, preclinical Alzheimer’s composite (z-composite); VLMT, German adaptation of Rey’s Auditory Verbal Learning Test (delayed verbal memory performance); ROCFT, Rey-Osterrieth Complex Figure Test (visuospatial delayed memory performance); BG PVS, total basal ganglia perivascular space volume (in mL); CSO PVS, total centrum semiovale perivascular space volume (in mL); WMH, total white matter hyperintensity volume (in mL); MTL DVR, , medial temporal lobe (MTL) tau load as distribution volume ratio (DVR); plasma pTau217 levels (in pg/ml) and the Aβ₁₋₄₂/ Aβ₁₋₄₀ ratio. All variables are shown as uncorrected raw values, except the PACC5 z-composite.

## Table S3

| **Outcome** | **N** | **M** | **SD** | **Median** | **IQR** |
| --- | --- | --- | --- | --- | --- |
| total GM volume | 263 | 584520 | 24005.2 | 585742.9 | 31671.9 |
| MTL thickness | 263 | 2.80 | 0.14 | 2.80 | 0.19 |
| bilat wHCV | 260 | 6935.03 | 597.86 | 6927.18 | 840.65 |
| bilat aHCV | 260 | 3546.81 | 424.38 | 3568.92 | 571.87 |
| bilat pHCV | 260 | 3388.21 | 330.91 | 3407.69 | 429.05 |
| left wHCV | 260 | 3437.58 | 313.96 | 3454.60 | 412.24 |
| left aHCV | 260 | 1732.86 | 217.34 | 1734.65 | 274.00 |
| left pHCV | 260 | 1704.72 | 176.39 | 1713.00 | 220.83 |
| right wHCV | 260 | 3497.44 | 317.66 | 3521.09 | 443.88 |
| right aHCV | 260 | 1813.95 | 239.79 | 1820.39 | 304.73 |
| right pHCV | 260 | 1683.49 | 170.56 | 1695.23 | 241.95 |
| BDNF | 89 | 2.70 | 0.36 | 2.72 | 0.56 |
| VEGF | 86 | 5.16 | 0.75 | 5.23 | 0.92 |
| Cathepsin-B | 87 | 4.74 | 0.54 | 4.80 | 0.86 |

Descriptive statistics of structural brain measures and plasticity-related factors. Uncorrected Raw values are presented as mean (M), standard deviation (SD), median and interquartile range (IQR). MTL thickness, medial temporal lobe thickness (in mm); total GMV volume, total gray matter volume; Volumetric measures for the anterior hippocampus (aHCV), posterior hippocampus (pHCV), and total hippocampal volume (wHCV) are shown for both hemispheres (bilateral), as well as for the left and right sides; BDNF, brain derived neurotrophic factor (ng/ml), VEGF,vascular endothelial growth factor (pg/ml) and Cathepsin B (ng/ml). All volumes are expressed in mm^3^ and corrected for total intracranial volume.

## Hypothesis 1: Association between Physical Fitness and Cognitive Performance

The associations between muscular composite variables (TUG, ASMM, and maximal handgrip strength) and cognitive performance were examined using separate regression models for each cognitive outcome. No significant associations with cognitive performance were observed (Table S4).

## Table S4

| Predictor | β | SE | p | p_adj | Predictor | β | SE | p | p_adj |
| --- | --- | --- | --- | --- | --- | --- | --- | --- | --- |
| **Model 1 - PACC5** | | | | | **Model 3 - VLMT** | | | | |
| Intercept | 1.178 | 0.425 | 0.006 | 0.021* | Intercept | 0.912 | 0.650 | 0.162 | 0.349 |
| TUG | −0.002 | 0.044 | 0.970 | 0.970 | TUG | 0.011 | 0.075 | 0.886 | 0.919 |
| Handgrip | −0.053 | 0.035 | 0.133 | 0.310 | Handgrip | −0.086 | 0.066 | 0.196 | 0.366 |
| ASMM | −0.019 | 0.044 | 0.667 | 0.812 | ASMM | −0.080 | 0.067 | 0.232 | 0.397 |
| Age | −0.036 | 0.005 | <0.001** | <0.001** | Age | −0.033 | 0.008 | <0.001** | <0.001** |
| Sex | 0.326 | 0.092 | <0.001** | <0.001** | Sex | 0.510 | 0.143 | <0.001** | <0.001** |
| Educ. years | 0.087 | 0.014 | <0.001** | <0.001** | Educ. years | 0.084 | 0.021 | <0.001** | <0.001** |
| **Model 2 - CERAD** | | | | | **Model 4 - RCFT** | | | | |
| Intercept | 0.658 | 0.313 | 0.036 | 0.101 | Intercept | 1.841 | 0.747 | 0.014 | 0.044* |
| TUG | 0.025 | 0.033 | 0.444 | 0.615 | TUG | 0.062 | 0.078 | 0.422 | 0.615 |
| Handgrip | −0.031 | 0.026 | 0.241 | 0.397 | Handgrip | 0.016 | 0.062 | 0.792 | 0.892 |
| ASMM | −0.019 | 0.032 | 0.556 | 0.708 | ASMM | −0.017 | 0.077 | 0.828 | 0.892 |
| Age | 0.001 | 0.004 | 0.827 | 0.892 | Age | −0.031 | 0.009 | <0.001** | <0.001** |
| Sex | −0.073 | 0.068 | 0.282 | 0.439 | Sex | −0.119 | 0.161 | 0.461 | 0.615 |
| Educ. years | −0.016 | 0.010 | 0.112 | 0.285 | Educ. years | 0.032 | 0.024 | 0.194 | 0.366 |

Multiple linear regression models, Hypothesis 1: assessing the effect of TUG (z-score and reverse-coded), ASMM (z-score), and maximal handgrip strength (z-score), age (in years), sex, and education years (covariates) on global cognitive performance measures (PACC5, CERAD) and delayed memory performance (VLMT, ROCFT) as dependent variables (total effect, mediation analysis). β, regression coefficient (slope); SE, standard error; p, unadjusted p-value; p_adj, FDR-corrected p-value (False Discovery Rate, Benjamini–Hochberg procedure); * p < 0.05; ** p < 0.001.

## Hypothesis 2: Physical Fitness and Resistance to Brain Pathology

In secondary analyses, individual components of the muscular capacity composite (TUG, ASMM, and maximal handgrip strength) were examined separately in relation to pathology markers (Table S5). Only the Timed-Up-and-Go test (TUG) was significantly associated with BG PVS volume (Fig. S1A and Table S5), indicating that better walking performance was associated with lower BG PVS volumes. To distinguish motor from executive contributions, the Trail Making Test ratio (TMT B/A), indexing executive control, was included as an additional covariate. TUG remained a significant predictor (β = −0.28, SE = 0.09, p = .002), whereas TMT B/A was not associated with BG PVS volume (β = −0.03, SE = 0.06, p = .632). In contrast, neither maximal handgrip strength nor ASMM was associated with BG PVS volume (Fig. S1B–C; Table S5) or any other pathology marker (all p_adjusted_ > .05).

## Table S5.

| **Predictor** | **β** | **SE** | **p** | **p_adj** | **Predictor** | **β** | **SE** | **p** | **p_adj** |
| --- | --- | --- | --- | --- | --- | --- | --- | --- | --- |
| **Model 1 - BG PVS** | | | | | **Model 4 - MTL Tau PET DVR** | | | | |
| (Intercept) | −3.210 | 0.848 | 0.000 | < 0.001** | (Intercept) | 0.986 | 0.078 | < 0.001** | < 0.001** |
| **TUG** | **−0.266** | **0.097** | **0.007** | **0.025*** | TUG | −0.001 | 0.010 | 0.925 | 0.960 |
| Handgrip | −0.082 | 0.071 | 0.253 | 0.462 | Handgrip | 0.004 | 0.006 | 0.464 | 0.629 |
| ASMM | −0.088 | 0.114 | 0.443 | 0.620 | ASMM | 0.002 | 0.010 | 0.824 | 0.911 |
| Age | 0.046 | 0.011 | < 0.001** | < 0.001** | Age | −0.001 | 0.001 | 0.401 | 0.602 |
| Sex | −0.013 | 0.221 | 0.953 | 0.960 | Sex | 0.023 | 0.019 | 0.229 | 0.437 |
| **Model 2 - CSO PVS** | | | | | **Model 5 - pTau217** | | | | |
| (Intercept) | −0.908 | 0.915 | 0.322 | 0.541 | (Intercept) | −3.417 | 0.370 | < 0.001** | < 0.001** |
| TUG | −0.174 | 0.105 | 0.098 | 0.257 | TUG | −0.080 | 0.042 | 0.059 | 0.177 |
| Handgrip | 0.060 | 0.077 | 0.436 | 0.620 | Handgrip | −0.002 | 0.031 | 0.960 | 0.960 |
| ASMM | −0.131 | 0.123 | 0.291 | 0.509 | ASMM | −0.063 | 0.050 | 0.211 | 0.437 |
| Age | 0.015 | 0.012 | 0.216 | 0.437 | Age | 0.018 | 0.005 | < 0.001** | < 0.001** |
| Sex | −0.215 | 0.238 | 0.368 | 0.572 | Sex | −0.204 | 0.095 | 0.033 | 0.107 |
| **Model 3 - WMH** | | | | | **Model 6 - Aβ42/40** | | | | |
| (Intercept) | −3.460 | 0.812 | < 0.001** | < 0.001** | (Intercept) | −2.129 | 0.121 | < 0.001** | < 0.001** |
| TUG | −0.167 | 0.093 | 0.074 | 0.207 | TUG | 0.006 | 0.014 | 0.695 | 0.834 |
| Handgrip | −0.062 | 0.068 | 0.364 | 0.572 | Handgrip | −0.003 | 0.010 | 0.737 | 0.852 |
| ASMM | 0.010 | 0.109 | 0.929 | 0.960 | ASMM | −0.011 | 0.016 | 0.492 | 0.646 |
| Age | 0.047 | 0.011 | 0.000 | < 0.001** | Age | −0.004 | 0.002 | 0.005 | 0.019* |
| Sex | 0.257 | 0.211 | 0.225 | 0.437 | Sex | 0.010 | 0.031 | 0.751 | 0.852 |
| **Model 7 - GFAP** | | | | |  |  |  |  |  |
| (Intercept) | 2.062 | 0.301 | < 0.001** | < 0.001** |  |  |  |  |  |
| TUG | 0.015 | 0.035 | 0.677 | 0.834 |  |  |  |  |  |
| Handgrip | 0.016 | 0.026 | 0.539 | 0.686 |  |  |  |  |  |
| ASMM | −0.055 | 0.041 | 0.178 | 0.437 |  |  |  |  |  |
| Age | 0.027 | 0.004 | < 0.001** | < 0.001** |  |  |  |  |  |
| Sex | 0.101 | 0.078 | 0.195 | 0.437 |  |  |  |  |  |

Multiple linear regression models, Hypothesis 2: assessing the effect of TUG (z-score and reverse-coded), ASMM (z-score), and maximal handgrip strength (z-score), age (in years), sex, and education years (covariates) on pathology markers. PVS, perivascular spaces; BG PVS, basal ganglia perivascular spaces; CSO PVS, centrum semiovale perivascular spaces; WMH, white matter hyperintensity; Tau, MTL DVR: medial temporal lobe mean distribution volume ratio; ptau217, phosphorylated tau at the 217th amino acid position; Aβ42/40, ratio of amyloid-β (Aβ) peptides Aβ1-42/ Aβ1-40; β, regression coefficient (slope); SE, standard error; p, unadjusted p-value; p_adj, FDR-corrected p-value (False Discovery Rate, Benjamini–Hochberg procedure); * p < 0.05;; ** p < 0.001.

**Fig. S1. Multiple linear regression plots illustrating the contribution of muscular fitness components to brain resistance**. Effect of TUG (timed-up-and-go-test) assessing physical performance; A), Handgrip strength (B), and ASMM (appendicular skeletal muscle mass; C) on BG PVS volumes (basal ganglia perivascular spaces, normalized for BG volume). The estimated regression line is displayed with its 95% confidence interval (light pink shaded area). β: regression slope (beta coefficient); SE: standard error; Adjusted p: FDR-corrected p-value (Benjamini–Hochberg procedure). * indicates Adjusted p < .05. All models were adjusted for age and sex.

## Hypothesis 4: Neurobiological Mechanisms Linking Physical Fitness and Cognitive Function

## Table S5.

| **Predictor** | **β** | **SE** | **p** | **p_adj** | **Predictor** | **β** | **SE** | **p** | **p_adj** |
| --- | --- | --- | --- | --- | --- | --- | --- | --- | --- |
| **Model 1 - wHC bilat** | | | | | **Model 6 - left pHC** | | | | |
| Intercept | 8,831 | 519 | < 0.001** | < 0.001** | Intercept | 1,942 | 147 | < 0.001** | < 0.001** |
| TUG | 80.45 | 56.46 | 0.155 | 0.252 | **TUG** | **34.398** | **16.006** | **0.033** | **0.088** |
| Handgrip | −47.69 | 42.42 | 0.262 | 0.390 | Handgrip | −9.066 | 12.025 | 0.452 | 0.565 |
| ASMM | −50.07 | 50.32 | 0.321 | 0.432 | ASMM | 22.312 | 14.265 | 0.119 | 0.208 |
| Age | −25.16 | 6.12 | < 0.001** | < 0.001** | Age | −5.879 | 1.736 | 0.001 | 0.004** |
| Sex | 20.53 | 106.81 | 0.848 | 0.860 | Sex | 120.330 | 30.278 | 0.000 | < 0.001*** |
| Educ. (years) | −7.52 | 16.59 | 0.651 | 0.711 | Educ. (years) | 8.018 | 4.705 | 0.090 | 0.166 |
| **Model 2 - aHC bilat** | | | | | **Model 7 - right wHC** | | | | |
| Intercept | 4,867 | 382 | <0.001** | <0.001** | Intercept | 4,485 | 279 | <0.001** | <0.001** |
| TUG | 28.59 | 41.55 | 0.492 | 0.584 | TUG | 30.60 | 30.41 | 0.315 | 0.432 |
| Handgrip | −33.39 | 31.22 | 0.286 | 0.409 | Handgrip | −19.83 | 22.84 | 0.386 | 0.510 |
| **ASMM** | **−86.77** | **37.03** | **0.020** | **0.058** | ASMM | −39.47 | 27.10 | 0.147 | 0.245 |
| Age | −12.35 | 4.50 | 0.007 | 0.022* | Age | −12.34 | 3.29 | < 0.001** | < 0.001** |
| Sex | −157.52 | 78.61 | 0.046 | 0.111 | Sex | −26.94 | 57.53 | 0.640 | 0.711 |
| Educ.(years) | −24.06 | 12.21 | 0.050 | 0.117 | Educ.(years) | −6.21 | 8.94 | 0.487 | 0.584 |
| **Model 3 - pHC bilat** | | | | | **Model 8 - right aHC** | | | | |
| Intercept | 3,963 | 277 | < 0.001** | < 0.001** | Intercept | 2,464 | 217 | < 0.001** | < 0.001** |
| TUG | 51.85 | 30.09 | 0.086 | 0.163 | TUG | 13.14 | 23.59 | 0.578 | 0.662 |
| Handgrip | −14.29 | 22.61 | 0.528 | 0.616 | Handgrip | −14.60 | 17.72 | 0.411 | 0.523 |
| ASMM | 36.70 | 26.82 | 0.172 | 0.274 | **ASMM** | **−53.86** | **21.02** | **0.011** | **0.033*** |
| Age | −12.80 | 3.26 | 0.000 | <0.001** | Age | −5.42 | 2.55 | 0.035 | 0.088 |
| Sex | 178.05 | 56.93 | 0.002 | 0.007** | Sex | −84.67 | 44.62 | 0.059 | 0.129 |
| Educ.(years) | 16.54 | 8.84 | 0.063 | 0.134 | Educ.(years) | −14.74 | 6.93 | 0.034 | 0.088 |
| **Model 4 - left wHC** | | | | | **Model 9 - right pHC** | | | | |
| Intercept | 4,345 | 272 | < 0.001** | < 0.001** | Intercept | 2,021 | 145 | < 0.001** | < 0.001** |
| TUG | 49.84 | 29.63 | 0.094 | 0.169 | TUG | 17.45 | 15.78 | 0.270 | 0.394 |
| Handgrip | −27.85 | 22.26 | 0.212 | 0.330 | Handgrip | −5.22 | 11.85 | 0.660 | 0.711 |
| ASMM | −10.60 | 26.41 | 0.688 | 0.730 | ASMM | 14.39 | 14.06 | 0.307 | 0.430 |
| Age | −12.81 | 3.21 | < 0.001** | < 0.001*** | Age | −6.92 | 1.71 | < 0.001** | < 0.001** |
| Sex | 47.47 | 56.06 | 0.398 | 0.516 | Sex | 57.72 | 29.86 | 0.054 | 0.122 |
| Educ. (years) | −1.30 | 8.71 | 0.881 | 0.881 | Educ. (years) | 8.52 | 4.64 | 0.067 | 0.138 |
| **Model 5 - left aHC** | | | | |  | | | | |
| Intercept | 2,402.89 | 196.40 | < 0.001** | < 0.001** |  |  |  |  |  |
| TUG | 15.45 | 21.33 | 0.470 | 0.577 |  |  |  |  |  |
| Handgrip | −18.79 | 16.03 | 0.242 | 0.368 |  |  |  |  |  |
| ASMM | −32.91 | 19.01 | 0.085 | 0.163 |  |  |  |  |  |
| Age | −6.93 | 2.31 | 0.003 | 0.010* |  |  |  |  |  |
| Sex | −72.85 | 40.36 | 0.072 | 0.144 |  |  |  |  |  |
| Educ.(years) | −9.32 | 6.27 | 0.138 | 0.236 |  |  |  |  |  |

Multiple linear regression models, Hypothesis 4: assessing the effect of TUG (z-score and reverse-coded), ASMM (z-score), and maximal handgrip strength (z-score), age (in years), sex and education years (covariates) on total, left and right hippocampal volume as dependent variable. wHCV, whole hippocampal volume [mm3]; aHCV, anterior hippocampal volume [mm3]; pHCV, posterior hippocampal volume [mm3]; β, beta coefficient (slope); SE, standard error; p, unadjusted p-value; p_adj, FDR- corrected (False Discovery Rate) p-value (Benjamini-Hochberg procedure); * p < 0.05; **p < 0.001.

## Table S7

| **Predictor** | **β** | **SE** | **p** | **Predictor** | **β** | **SE** | **p** |
| --- | --- | --- | --- | --- | --- | --- | --- |
| **Model - GMV** | | | | **Model - MTL thickness** | | | |
| Intercept | 692,138 | 17,418 | <0.001** | Intercept | 3.121 | 0.114 | < 0.001** |
| **TUG** | **5,808** | **1,907** | **0.003*** | **TUG** | **0.032** | **0.013** | **0.011*** |
| Handgrip | 372 | 1,429 | 0.794 | Handgrip | −0.004 | 0.009 | 0.644 |
| **ASMM** | **−3,852** | **1,694** | **0.024*** | ASMM | −0.005 | 0.011 | 0.638 |
| Age | −1,587 | 205 | < 0.001** | Age | −0.004 | 0.001 | 0.001* |
| Sex | 1,135 | 3,594 | 0.752 | Sex | 0.042 | 0.024 | 0.073 |
| Education (years) | 305 | 561 | 0.586 | Education (years) | −0.002 | 0.004 | 0.581 |

Multiple linear regression models, Hypothesis 4: assessing the effect of TUG (z-score and reverse-coded), ASMM (z-score), and maximal handgrip strength (z-score), age (in years), sex and education years (covariates) on total GMV, gray matter volume [mm3] and MTL thickness, medial temporal lobe thickness (in mm); β, beta coefficient (slope); SE, standard error; p, unadjusted p-value; p_adj, FDR- corrected (False Discovery Rate) p-value (Benjamini-Hochberg procedure); * p < 0.05; ** p < 0.001.

## Table S8

| **Predictor** | **β** | **SE** | **p** | **p_adj** | **Predictor** | **β** | **SE** | **p** | **p_adj** |
| --- | --- | --- | --- | --- | --- | --- | --- | --- | --- |
| **Model 1 - BDNF** | | | | | **Model 2 - VEGF** | | | | |
| Intercept | 3.294 | 0.550 | <0.001** | <0.001** | Intercept | 5.074 | 1.179 | <0.001** | <0.001** |
| TUG | 0.017 | 0.059 | 0.770 | 0.935 | TUG | 0.011 | 0.126 | 0.928 | 0.935 |
| Handgrip | −0.006 | 0.051 | 0.907 | 0.935 | Handgrip | 0.070 | 0.109 | 0.519 | 0.935 |
| ASMM | −0.046 | 0.068 | 0.500 | 0.935 | ASMM | 0.187 | 0.146 | 0.202 | 0.654 |
| Age | −0.010 | 0.006 | 0.133 | 0.559 | Age | 0.001 | 0.014 | 0.935 | 0.935 |
| Sex | 0.051 | 0.134 | 0.704 | 0.935 | Sex | 0.335 | 0.288 | 0.249 | 0.654 |
| Education (years) | 0.006 | 0.019 | 0.774 | 0.935 | Education (years) | −0.009 | 0.043 | 0.841 | 0.935 |
| **Model 3 - Cathepsin B** | | | | |  |  |  |  |  |
| Intercept | 4.215 | 0.821 | <0.001** | <0.001** |  |  |  |  |  |
| TUG | −0.008 | 0.087 | 0.932 | 0.935 |  |  |  |  |  |
| Handgrip | −0.137 | 0.076 | 0.075 | 0.394 |  |  |  |  |  |
| ASMM | −0.122 | 0.102 | 0.234 | 0.654 |  |  |  |  |  |
| Age | 0.006 | 0.010 | 0.511 | 0.935 |  |  |  |  |  |
| Sex | −0.117 | 0.201 | 0.560 | 0.935 |  |  |  |  |  |
| Education (years) | 0.009 | 0.030 | 0.766 | 0.935 |  |  |  |  |  |

Multiple linear regression models, Hypothesis 4: assessing the effect of TUG (z-score and reverse-coded), ASMM (z-score), and maximal handgrip strength (z-score), age (in years), sex and education years (covariates) on BDNF, brain derived neurotrophic factor (ng/ml), VEGF, vascular endothelial growth factor (pg/ml) and Cathepsin B (ng/ml); β, beta coefficient (slope); SE, standard error; p, unadjusted p-value; p_adj, FDR- corrected (False Discovery Rate) p-value (Benjamini-Hochberg procedure); * p < 0.05; ** p < 0.001.
